# Supplementary material for: Evolution of Antibiotic Resistance and the Relationship between the Antibiotic Resistance Genes and Microbial Compositions under Long-Term Exposure to Tetracycline and Sulfamethoxazole
Source: Int J Environ Res Public Health. 2019 Nov 25;16(23):4681. doi: 10.3390/ijerph16234681 (PMC6926690; doi:10.3390/ijerph16234681)
Supplement: Supplementary file 1 [file ijerph-16-04681-s001.pdf]

Supplementary

Table S1 Conditions of antibiotics analysis by high performance liquid chromatography

Analyte	Tetracycline	Sulfanilamide
Mobile phase	A: 0.1% formic acid; B: Acetonitrile. The gradient solvent was as follows: B = 5% (0 min), 5% (0.5 min), 20% (3 min), 70% (3.5 min), 95% (8 min) and 5% (12 min).	A: 0.1% formic acid; B: Acetonitrile. The gradient solvent was as follows: B = 10% (0 min), 10% (1 min), 30% (3 min), 65% (5 min), 70% (10 min) and 10% (12 min).
sample injection volume（μL）	10	10
flow rate（ml/min）	0.35	0.80
Column temperature (℃)	35	35
standard curve	y = 229.86x - 2.22	y = 649.82x - 56.81
r2	0.9992	0.9943

Table S2. The relative abundances (gene copies of ARGs normalized to the gene copies of 16S rRNA) of six tetracycline (TC) resistance genes (*tetA, tetC, tetL, tetO, tetW* and *tetX*) and three sulfonamide resistance genes (*sul1, sul2* and *sul3*) as well as the integrase gene of class 1 integrons (*intI1*) in different phases in system A (control system) and system B (system exposed to antibiotics).

Operational phases	Tetracycline resistance genes											
	tetA		*tetC*		*tetL*		*tetO*		*tetW*		*tetX*	
	A	B	A	B	A	B	A	B	A	B	A	B
P1	1.41E-04	5.53E-04	2.69E-03	5.01E-03	8.14E-06	8.01E-04	3.26E-06	6.23E-06	1.56E-04	2.46E-04	4.82E-04	7.22E-04
P2-1	1.14E-03	6.15E-03	3.35E-03	2.12E-02	6.00E-04	4.57E-04	5.17E-06	2.22E-05	1.01E-04	2.16E-04	7.72E-04	1.51E-03
P2-2	2.41E-03	9.23E-03	1.12E-02	3.17E-02	1.22E-04	3.34E-05	2.38E-06	2.92E-05	1.53E-04	8.63E-05	2.34E-03	6.62E-04
P2-3	4.67E-03	2.63E-02	8.74E-03	1.69E-02	8.34E-05	2.63E-05	1.95E-05	1.17E-05	7.72E-05	1.73E-04	1.70E-03	7.67E-04
P3	4.64E-03	1.29E-02	3.39E-04	2.40E-02	1.91E-04	7.70E-05	1.16E-05	1.27E-04	2.09E-04	4.80E-04	3.20E-04	1.16E-03
P4-1	9.59E-03	5.28E-03	2.73E-03	7.74E-02	2.14E-04	1.96E-03	2.53E-05	2.87E-05	2.53E-04	4.64E-04	1.84E-03	3.44E-03
P4-2	9.05E-03	6.02E-03	3.46E-03	6.17E-02	9.88E-05	1.63E-03	1.51E-05	9.45E-06	1.42E-04	7.08E-04	3.56E-03	9.32E-03
P4-3	3.50E-03	2.68E-03	1.73E-03	5.77E-02	6.78E-05	7.30E-03	1.05E-05	7.59E-06	4.18E-05	1.83E-04	4.51E-03	5.85E-03
Max	9.59E-03	2.63E-02	1.12E-02	7.74E-02	6.00E-04	7.30E-03	2.53E-05	1.27E-04	2.53E-04	7.08E-04	4.51E-03	9.32E-03
Mean	4.39E-03	8.64E-03	4.28E-03	3.69E-02	1.73E-04	1.54E-03	1.16E-05	3.03E-05	1.42E-04	3.20E-04	1.94E-03	2.93E-03
Min	1.41E-04	5.53E-04	3.39E-04	5.01E-03	8.14E-06	2.63E-05	2.38E-06	6.23E-06	4.18E-05	8.63E-05	3.20E-04	6.62E-04
SD	3.43E-03	8.06E-03	3.71E-03	2.55E-02	1.85E-04	2.44E-03	8.11E-06	4.03E-05	6.86E-05	2.10E-04	1.49E-03	3.15E-03
p	.195		.010*		.166		.237		.030*		.250	

Operational phases	Sulfonamide resistance genes and the integrase gene of class 1 integron							
	sul1		*sul2*		*sul3*		*intIl*	
	A	B	A	B	A	B	A	B
P1	1.42E-02	1.92E-02	1.08E-02	1.03E-02	3.49E-06	5.90E-06	7.82E-03	7.91E-03
P2-1	1.80E-02	5.54E-02	1.69E-02	2.01E-02	1.03E-05	7.72E-05	7.12E-04	6.21E-04
P2-2	5.66E-02	7.14E-02	1.35E-02	1.50E-02	2.59E-05	2.74E-05	1.92E-03	1.14E-03
P2-3	2.59E-02	6.57E-02	1.51E-03	8.04E-03	3.20E-06	4.78E-06	3.07E-03	1.47E-03
P3	9.63E-03	4.87E-02	6.34E-03	2.47E-02	1.89E-06	1.23E-05	1.94E-04	2.35E-03
P4-1	3.50E-02	8.59E-02	1.62E-02	2.71E-02	7.33E-06	2.23E-05	7.07E-04	1.29E-03
P4-2	7.00E-02	2.23E-01	1.37E-02	5.80E-02	1.34E-05	6.71E-05	6.09E-04	3.20E-03
P4-3	8.11E-02	1.88E-01	1.58E-02	5.17E-02	8.47E-06	6.82E-05	7.33E-04	6.32E-04
Max	8.11E-02	2.23E-01	1.69E-02	5.80E-02	2.59E-05	7.72E-05	7.82E-03	7.91E-03
Mean	3.88E-02	9.46E-02	1.18E-02	2.69E-02	9.25E-06	3.57E-05	1.97E-03	2.33E-03
Min	9.63E-03	1.92E-02	1.51E-03	8.04E-03	1.89E-06	4.78E-06	1.94E-04	6.21E-04
SD	2.71E-02	7.16E-02	5.41E-03	1.85E-02	7.79E-06	3.02E-05	2.54E-03	2.42E-03
p	.015*		.039*		.035*		.498	

Unit, gene copies /16S Rrna; A = control system; B = system exposed to antibiotics; Max, maximum; Min, minimum; SD, standard deviation; Asterisks (*) represent significant differences ($p<0.05$); P1 = operational phase 1 (no antibiotic addition, days 0–40); P2 = operational phase 2 (addition of 5 $mg \cdot L^{-1}$ TC in system B, days 41–200); P3 = operational phase 3 (recovery phase, no antibiotic addition, days 201–240); P4 =

operational phase 4 (addition of 5mg·L^{-1} TC and 1 mg·L^{-1} sulfamethoxazole in system B, days 241–420).

Table S3. The abundance of dominant bacterial community at the genus level in the anoxic-aerobic systems (A = control system; B = system exposed to antibiotics) based on the high-throughput genetic sequencing.

	A1	B1	A100	B100	A200	B200	A240	B240	A330	B330	A420	B420
Sphaerotilus	0.03	0.14	0.25	1.71	34.33	31.13	0.59	0.56	5.70	1.81	1.64	0.42
Dechloromonas	9.54	6.91	26.90	21.82	0.29	0.83	1.15	1.36	0.67	8.14	5.05	25.24
Rheinheimera	0.10	0.43	2.34	9.38	34.72	16.09	0.91	1.13	0.25	0.09	0.03	0.01
Aeromonas	4.74	7.83	1.25	2.13	0.16	7.29	0.38	6.34	1.22	0.65	0.25	0.24
Flavobacterium	0.00	0.25	5.22	6.37	7.51	2.45	1.36	1.03	0.75	4.31	0.91	1.40
Hydrogenophaga	3.66	1.77	1.46	5.22	2.86	6.16	0.01	0.04	0.37	0.75	0.93	1.07
Thiothrix	0.01	0.00	12.78	2.48	0.23	0.17	0.11	0.12	3.46	24.09	1.40	5.50
Thauera	6.59	7.38	0.00	0.00	0.00	0.00	2.07	1.33	0.04	0.01	0.66	0.84
Azospira	0.10	0.23	6.67	5.89	0.06	0.11	0.01	0.01	3.07	0.26	0.75	0.34
Undibacterium	0.00	0.04	0.95	1.70	0.06	7.49	1.34	0.95	0.04	0.47	0.00	0.00
Tabrizicola	1.62	2.13	1.33	3.73	0.11	0.15	0.00	0.00	0.00	0.00	0.00	0.00
Zoogloea	3.18	4.30	0.04	0.27	0.05	0.12	1.63	1.64	1.81	0.06	0.00	0.00
Cloacibacterium	0.49	1.11	0.55	3.81	0.15	0.91	0.06	0.30	0.02	0.21	0.00	0.00
Rhodobacter	1.42	2.90	0.37	0.46	0.96	0.78	0.11	0.08	2.60	0.35	3.11	0.65
Nitrospira	3.01	3.40	0.07	0.04	0.01	0.01	1.83	1.09	0.14	0.05	0.27	0.13
Ferruginibacter	2.66	2.56	0.00	0.00	0.00	0.00	8.17	7.14	0.19	0.02	0.00	0.00
Brevundimonas	0.21	0.21	4.25	0.58	0.02	0.02	0.00	0.03	0.02	0.17	0.03	0.03

Crocinitomix	0.22	0.30	0.30	0.10	0.29	5.31	0.27	0.39	0.00	0.01	0.06	0.04
Sediminibacterium	0.00	0.00	0.06	0.38	0.27	3.88	0.10	0.67	5.32	1.52	2.98	0.82
Chitinophaga	0.00	0.00	4.50	0.35	0.02	0.05	0.02	0.03	0.29	0.22	0.23	0.15
Pseudomonas	0.00	0.00	0.00	0.39	0.02	0.04	1.57	5.76	0.11	0.37	0.61	0.95
Accumulibacter	0.01	0.00	0.22	0.13	0.01	0.10	0.02	0.01	2.96	5.39	5.25	9.57
Phaeodactylibacter	0.08	0.14	3.82	1.57	0.02	0.05	0.36	0.25	0.73	3.81	2.43	1.29
Thermomonas	0.92	0.59	0.00	0.00	0.00	0.00	0.55	0.40	2.64	3.60	0.88	1.79
Bdellovibrio	0.43	0.21	1.05	0.13	0.01	0.34	0.50	0.38	0.51	3.17	0.12	1.34
Runella	0.00	0.02	1.06	1.93	0.33	1.21	0.01	0.01	2.08	3.16	2.60	2.64
Haliscomenobacter	1.57	1.59	0.13	0.05	0.01	0.02	0.52	0.31	3.11	1.11	4.40	1.55
Emticicia	0.00	0.00	0.82	0.29	0.68	1.56	0.02	0.01	0.01	0.20	0.14	0.07
Terrimonas	1.27	1.45	0.00	0.00	0.00	0.00	2.03	1.69	0.22	0.32	0.09	0.13
Arcobacter	0.67	0.00	0.01	0.09	0.02	0.00	1.03	2.03	0.13	0.04	0.01	0.01
others	57.46	54.07	23.57	29.00	16.82	13.73	73.25	64.91	61.56	35.65	65.19	43.80

A1, B1, A100, B100, A200, B200, A240, B240, A330, B330, A420, B420, is corresponding to the operational day of control system A and antibiotics exposure system B on day 1, 100, 200, 240, 330 and 420, respectively.

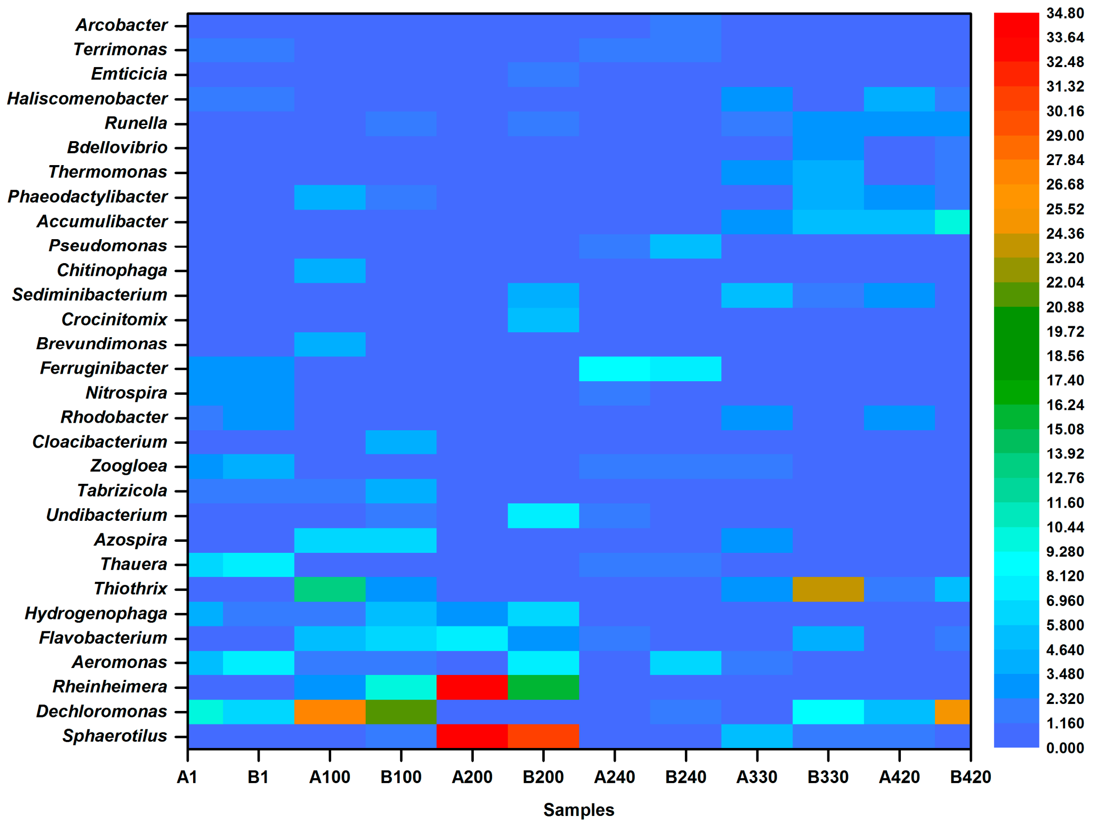

Fig. S1 The compositions of dominant bacterial community at the genus level in the anoxic-aerobic systems (A = control system; B = system exposed to antibiotics) based on the high-throughput genetic sequencing. The color intensity shows the relative abundance of each genus as the

color key indicates at the right of the figure. (A1, B1, A100, B100, A200, B200, A240, B240, A330, B330, A420, B420, is corresponding to the operational day of control system A and antibiotics exposure system B on day 1, 100, 200, 240, 330 and 420, respectively).
